# Mathematical modeling of the relocation of the divalent metal transporter DMT1 in the intestinal iron absorption process

**DOI:** 10.1371/journal.pone.0218123

**Published:** 2019-06-10

**Authors:** Layimar Cegarra, Andrea Colins, Ziomara P. Gerdtzen, Marco T. Nuñez, J. Cristian Salgado

**Affiliations:** 1 Laboratory of Process Modeling and Distributed Computing, Department of Chemical Engineering, Biotechnology and Materials, University of Chile, Santiago, Chile; 2 Centre for Biotechnology and Bioengineering, Department of Chemical Engineering, Biotechnology and Materials, University of Chile, Santiago, Chile; 3 Iron and Biology of Aging Laboratory, Department of Biology, Faculty of Sciences, University of Chile, Santiago, Chile; Northeastern University, UNITED STATES

## Abstract

Iron is essential for the normal development of cellular processes. This metal has a high redox potential that can damage cells and its overload or deficiency is related to several diseases, therefore it is crucial for its absorption to be highly regulated. A fast-response regulatory mechanism has been reported known as mucosal block, which allows to regulate iron absorption after an initial iron challenge. In this mechanism, the internalization of the DMT1 transporters in enterocytes would be a key factor. Two phenomenological models are proposed for the iron absorption process: *DMT1’s binary switching mechanism model* and *DMT1’s swinging-mechanism model*, which represent the absorption mechanism for iron uptake in intestinal cells. The first model considers mutually excluding processes for endocytosis and exocytosis of DMT1. The second model considers a Ball’s oscillator to represent the oscillatory behavior of DMT1’s internalization. Both models are capable of capturing the kinetics of iron absorption and represent empirical observations, but the *DMT1’s swinging-mechanism model* exhibits a better correlation with experimental data and is able to capture the regulatory phenomenon of mucosal block. The *DMT1 swinging-mechanism model* is the first phenomenological model reported to effectively represent the complexity of the iron absorption process, as it can predict the behavior of iron absorption fluxes after challenging cells with an initial dose of iron, and the reduction in iron uptake observed as a result of mucosal block after a second iron dose.

## Introduction

Iron is the most abundant trace metal in mammalian species. It is essential for normal cellular and enzymatic functions due to its ability to cycle between two oxidation states: ferrous (*Fe*^2+^) and ferric (*Fe*^3+^) [1, 2]. This metal is required for oxygen transport to tissues, energy metabolism, cellular respiration and DNA synthesis [3–5]. Dietary iron exists in heme (10%) and non-heme or ionic (90%) forms [6]. Heme iron is a *Fe*^3+^–protoporphyrin IX complex, found in foods of animal origin in the form of hemoglobin or myoglobin; ionic iron is found in foods of plant origin, cereals and some foods of animal origin [7].

At physiological pH, ferrous iron is rapidly oxidized to the insoluble ferric form (*Fe*^3+^) [8], which is why cells require carrier proteins that allow iron transport and ensure its bioavailability [9]. On the other hand, iron has a high redox potential and can catalyze the Haber-Weiss reaction to generate hydroxyl radicals, which in turn can damage proteins, DNA and lipids [5, 10, 11]. Therefore, as *Fe*^3+^ can be damaging to cells, a specific and tightly regulated process controls the uptake, transport and storage of this metal [1].

A well-nourished average adult human has a total of 3–5 g of iron [4, 5]. About 65–75% of the body’s iron is found in hemoglobin of erythrocytes, 10% is in myoglobin of striated muscle, 10–20% associated to ferritin in the liver, 0.1% bound to transferrin in the bloodstream and the rest is distributed in other tissues [12, 13]. About 1–2 mg of iron is lost daily predominantly through desquamation of epithelial cells in the digestive tract and skin, minor blood loss, sweat and urine; in steady state the body compensates this loss through intestinal iron absorption [5, 9, 14, 15]. Erythrocytes synthesis requires 20–30 mg of iron and non-erythroid cells approximately 5 mg of iron per day [5, 9]. Macrophages can phagocyte senescent or damaged erythrocytes, extract their heme iron and recycle it to the extracellular fluid and plasma; in this process, the amount of iron necessary for daily erythropoiesis is recovered [14].

Both iron deficiency and iron overload are related to several diseases [9]. In recent years, several studies have been conducted to investigate the relationship between iron accumulation and neurodegenerative diseases such as Alzheimer, Huntington’s and Parkinson’s disease, carcinogenesis, sarcopenia [16, 17]. However, it has not been established whether iron accumulation is a symptom or a cause of these diseases [1]. On the other hand, iron deficiency impairs endurance capacity, immune function, thermoregulation, cognition, and restless leg syndrome [18, 19]. Hence a better understanding of the iron transport and homeostasis mechanisms can help deepen our knowledge of these diseases [5].

There is no known mechanism of iron excretion in mammals; therefore, the control of intestinal absorption of this metal from the duodenum in response to cellular iron requirements and availability is crucial for iron homeostasis both at the systemic and cellular levels [5, 20, 21].

**Fig 1:**
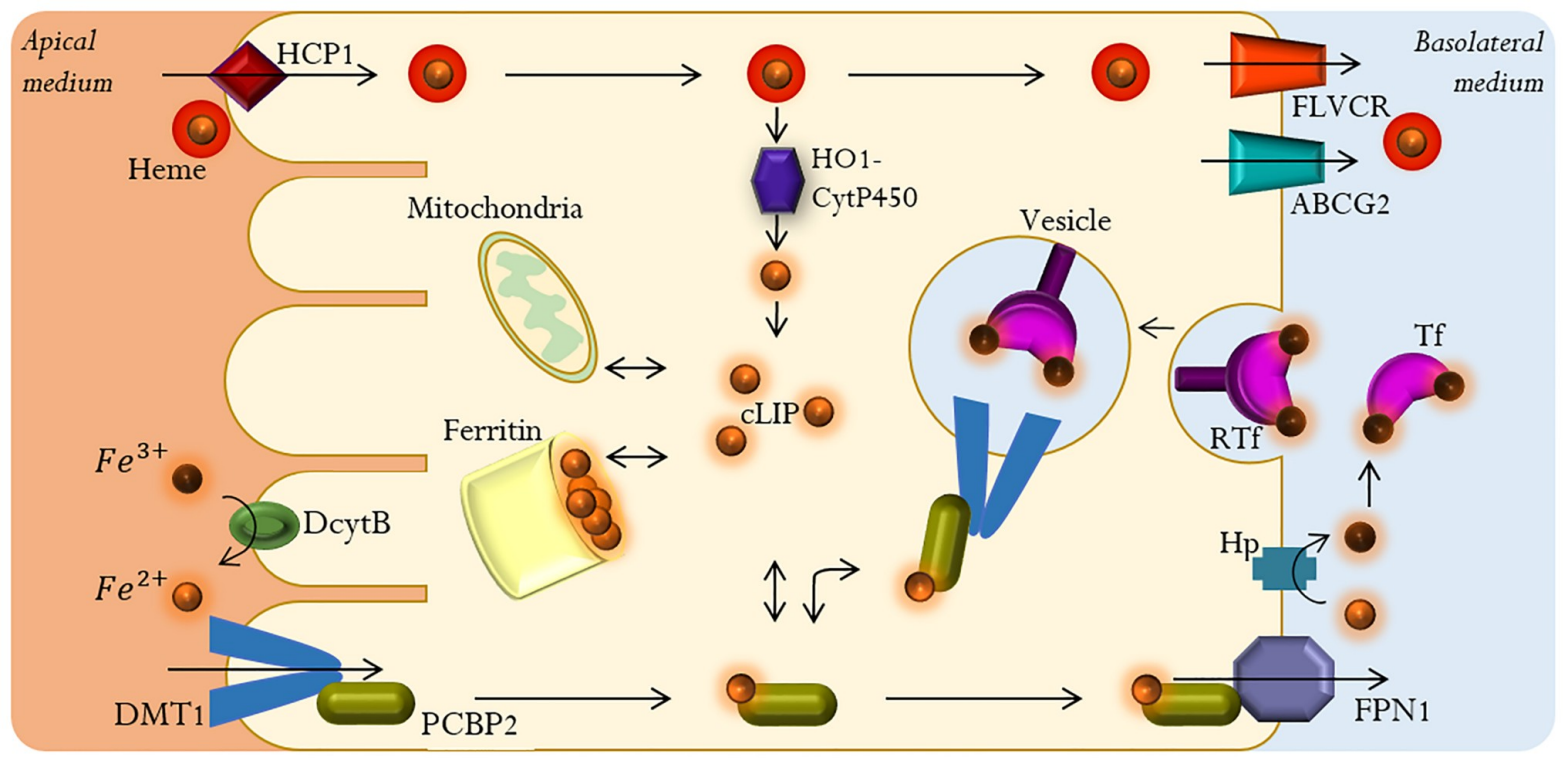
Main components of the intestinal iron absorption process.

In the absorption process iron is transported from the gut into the bloodstream. This process takes place predominantly in the proximal portion of the duodenum and upper jejunum, where enterocytes have a microvillous brush border at the apical surface in order to maximize absorptive surface area [20, 22]. Fig 1 shows the main components of the iron absorption process.

The absorption of iron in its heme form may involve heme carrier protein 1 (HCP1) [1]. It is known that once heme iron is internalized it is catabolized by a multi-enzyme complex HO1-CytP450 (cytosolic Heme Oxygenase-1/NADPH-Cytochrome P450 Reductase) into biliverdin and carbon monoxide, releasing *Fe*^2+^ [12, 23]. It has been proposed that the recently identified heme transport proteins of the FLVCR group (feline leukemia virus subgroup C receptor) and ABCG2 (ATP binding cassette protein G2), transport excess heme iron directly into the bloodstream [5, 24].

Dietary ionic iron *Fe*^3+^ is reduced to *Fe*^2+^ prior being absorbed by the gut, by the action of the Duodenal cytochrome B (DcytB), a ferric reductase localized on the brush border membrane (BBM) of the duodenum [12, 20, 25]. Once in its reduced form iron is transported across the BBM and into the cell by the Divalent Metal Transporter 1 (DMT1), an iron symporter that cotransports protons and *Fe*^2+^ [4, 20, 26, 27]. DMT1 can also transport other divalent metal cations (*Mn*^2+^, *Co*^2+^, *Zn*^2+^, *Cu*^2+^, *Pb*^2+^) [20]. This transporter is located in the apical membrane of enterocytes, where it mediates the uptake of *Fe*^2+^, and in the late endosomal membranes of all other cells types where it allows the transport of endocyted *Fe*^2+^ into the cytoplasm [12, 28]. Given the dependence of the transport process with proton concentration, treatment with antacids interferes with iron absorption as the availability of protons decreases [20, 26]. However, it has been reported that this transport can also occur in an H^+^ uncoupled manner [27]. An alternative transporter in cellular iron uptake has been reported, Zrt-like Irt-like protein 14 (Zip-14). The optimal pH for this alternative transporter is 7.5, hence its activity is thought to be suppressed in the duodenum where acidic conditions are found [29].

Depending on the organism’s systemic requirements, after entering the enterocyte *Fe*^2+^ can either be stored in ferritin or transferred into the bloodstream by ferroportin 1 (FPN1), the sole known cellular iron exporter, where it is oxidized from *Fe*^2+^ to *Fe*^3+^ by hephaestin (Hp), a multicopper oxidase [20, 25]. Iron can also become part of the cytosolic labile iron pool (cLIP), a pool of redox-active chelatable iron that represents *c.a.* 3% of the total cellular iron [30, 31]. *Fe*^3+^ can circulate in the bloodstream bound to transferrin (Tf), a glycoprotein with two binding sites for *Fe*^3+^ that can enter cells that bear specific transferrin receptors (RTf) through a receptor-mediated endocytosis process [1, 5, 12, 23]. Both ferritin and transferrin can sequester iron to maintain it in a nonreactive form [20, 25]. Generally, 30–40% of Tf is saturated with iron. When Tf’s saturation capacity is exceeded, non-transferrin-bound-iron (NTBI) is generated, which can cause cellular damage due to its unlimited redox activity as free *Fe*^3+^ [5, 20].

Given the crucial role of iron in cellular processes, its potentially damaging effects for the cell and the lack of an iron excretion mechanism, iron absorption and homeostasis must be tightly controlled processes. There are four known mechanisms that regulate iron absorption: systemic, translational, transcriptional and mucosal block, with response times varying from minutes to days after an iron challenge [32]. Regulatory mechanisms of this system operate at different scales of time and space. In particular, the mucosal block is a fast-response endocytic mechanism that has been described as the ability of an initial dose of ingested iron to block the absorption of a second dose [32, 33]. This mechanism allows enterocytes to modulate iron absorption after an initial iron challenge.

The iron transport process through the enterocyte is not fully understood. It has been reported that iron feeding (both ionic forms) induces internalization of DMT1 in the intestinal epithelial cells, from the BBM into vesicles within the intracellular compartment [25, 29, 34, 35], and translocation of Hp and FPN1 from the sub-apical compartment to the basal lateral membrane (BLM) [35, 36]. Once in the enterocyte, intracellular iron can be transported through an endocytic process where BMM-derived vesicles containing DMT1 are fused with BLM-derived vesicles containing apo-transferrin (apo-Tf). Iron is then bound to Tf where it can be exported to the bloodstream [25, 28, 32, 34]. In addition, an iron chaperone protein, poly(rC)-binding protein 2 (PCBP2), has recently been identified [37]. Iron transported by DMT1 can be transferred directly to PCBP2, which can then deliver it to the appropriate cellular site or donate it to FPN1. Therefore, PCBP2 can modulate the export of cellular iron [37].

Characterization of early iron exposure absorption fluxes *in vitro* requires controlled experimental conditions in a model cell. The human colon carcinoma cell line Caco-2 is the most accepted *in vitro* absorption model for the intestinal epithelial transport [38, 39]. These cells form a tight differentiated monolayer of mature intestinal enterocytes and allow the study of intestinal absorption mechanisms [38, 40]. The findings of Núñez *et al.* show that Caco-2 cells have bidirectional iron fluxes mediated by DMT1 and FPN1 in both, the apical and basolateral membranes [35].

The iron absorption process requires the coordinated operation of a series of biological mechanisms that interact in a highly complex manner. A deep knowledge of the interactions between these mechanisms would contribute to a better understanding on how diseases and disorders associated to the iron absorption system are triggered. Given the high complexity of this system, described above, we propose the use of mathematical modeling to establish an analytical framework for the description and analysis of the key elements in these processes and the interactions among them that are relevant for recovering the observed experimental behavior.

Previously, a mathematical model of systemic iron metabolism that comprises a set of iron pools within the body (iron in: plasma, circulating red cells, mucosal, parenchymal and reticuloendothelial cells) was proposed to simulate iron metabolism behavior under different therapeutical treatments [41]. Later, a multicompartment model was developed to describe the physiological process of intestinal iron absorption and plasma iron kinetics in normal dogs [42]. The model of Lao and Kamei, was improved including the liver as a key site of iron regulation, to study the mechanism of iron homeostasis [43]. Recently, a mathematical model which quantitatively describes systemic iron metabolism incorporating organ iron pool dynamics as well as regulation by the hepcidin/ferroportin system was proposed; this model also considers iron uptake saturation [44]. Parmar and Mendes presented a computational model of systemic iron homeostasis in a mouse. The model is capable of explaining iron distribution for a wide range of total body iron concentrations and can represent iron-related diseases through regulatory mechanisms [45]. All these models focus on iron metabolism at a systemic level. We developed the first iron metabolism model at a cellular level to study the dynamics of iron storage in ferritin during the process of intestinal iron absorption, considering a discrete population of ferritin species defined by their respective iron content and their main reactions [46]. To study the short-term effect of iron exposure in iron absorption fluxes we proposed a method for developing mathematical models for complex systems, based on a genetic algorithm (genetic programming). Even though Michaelis–Menten and Hill kinetics are the simplest way to characterize saturable uptake of nutrients in cell culture for single substrate—single product settings [47, 48], we showed in a previous work that these classic models cannot capture the key characteristics of the iron absorption process as they cannot represent more complex mechanisms that take place in this system [49]. The model developed by Colins *et al.* was able to capture the complex non-linear dynamics observed experimentally using a genetic algorithm methodology [49]. However, it is difficult to provide a biological interpretation for this specific model in terms of the relevant phenomena involved in iron absorption and its regulation. The model also lacks the flexibility necessary to be easily expanded.

The kinetic mechanism of an enzyme can be represented as a cycle formed by a finite number of discrete states. Transitions between states can describe interactions with ligands, substrates, products or conformational changes [50]. Mackenzie *et al.* proposed an eight-state mechanism to describe the DMT1 transport system. This mechanism represents both the simultaneous H^+^-coupled *Fe*^2+^ transport and uncoupled fluxes of H^+^ or *Fe*^2+^ mediated by DMT1 (see Fig 8 in [27]).

In this paper we propose a state based phenomenological model that takes into account the main biological components of this system, in order to the mechanistic complexities that have not been accounted for in mathematical models until now, to study the intestinal iron absorption process in Caco-2 cells. The model considers a description of DMT1 states and its internalization, in order to capture the complex iron uptake dynamics observed experimentally.

## Materials and methods

### *In vitro* procedure

#### Cell lines and culture medium

Caco-2 cells [HTB-37, American Type Culture Collection (ATCC), Rockville, MD] were cultured in Dulbecco’s modified Eagle’s medium (DMEM) supplemented with 10% fetal bovine serum (FBS, Invitrogen-Gibco Life Technologies) and 1% antibiotic and antifungal solution, at 37 °C with 5% CO_2_-95% air. Cells were grown for 17 to 20 days in 12 mm diameter bicameral inserts (Corning Costar). Inserts with transepithelial electrical resistance (TEER) threshold above 240 Ωcm^2^ were used in the experiments [39, 51]; to ensure the differentiation of tight junctions, which is indicative of the integrity and functionality of the monolayer [38]. Culture media was replaced every other day. To limit the variability of the experiments, cell passage number range was maintained below 15.

#### Measurement of iron uptake

Fully differentiated insert-grown cells were incubated overnight in DMEM with 2% FBS [52]. Cells were challenged with an iron concentration in the apical media of 20 *μ*M ^55^FeCl_3_–ascorbate (1:20, mol:mol) in DMEM. Experiments were performed in triplicate between 3 and 15 minutes at 37 °C. Uptake was considered as the total radioactivity in the cells plus the basolateral medium after incubation. Fe–ascorbate was used to avoid a possible interference of Dcytb ferrireductase with the uptake process [49].

#### Measurement of iron uptake after a second iron challenge

Fully differentiated insert-grown cells were incubated overnight in DMEM with 2% FBS; subsequently they were exposed to an initial iron challenge in the apical media with a concentration of 20 *μ*M ^56^FeCl_3_–ascorbate (1:20, mol:mol) in DMEM. Cells were incubated for 15 minutes at 37 °C, placed on ice to halt all cellular processes, and then washed three times with cold phosphate-buffered saline (PBS). Cells were then exposed to a second iron challenge in the apical media, with a concentration of 20 *μ*M ^55^FeCl_3_–ascorbate (1:20, mol:mol) in DMEM. Samples were analyzed after incubation between 3 and 15 minutes at 37 °C. All experiments were performed in triplicate. Uptake for the second challenge was considered as the total radioactivity in the cells plus the basolateral medium over time (^55^Fe and ^56^Fe), minus the radioactivity associated to the first challenge.

### *In silico* procedure

#### Phenomenological models

The iron transport process through the enterocyte is highly complex and, to date, not fully understood. We consider the following assumptions that simplify its representation while maintain key components:

1. *FPN1 activity is considered as not significant during the first 30 minutes*: the apical iron efflux through FPN1 in Caco-2 cells incubated with ^55^Fe–ascorbate, is very low [35]. In addition, at the beginning of the experiments there is no ^55^Fe in the basolateral medium, which can also be linked to a reduce FPN1 activity.
2. *Cell-to-apical iron fluxes due to transferrin activity are not considered*: Tf content in cells was reduced to a minimum by incubating cells in low serum media before experiments and performing experiments in serum-free media [52].
3. *Dcytb activity can be neglected*: In all experiments iron remains in its ferrous form due to the presence of ascorbic acid in the apical media.
4. *The mechanisms of systemic, transcriptional and translational regulation do not affect the system*: given the experimental time-scale, the effect of other regulation mechanisms is not considered as their response time ranges from hours to days after an iron challenge.
5. *Iron uptake by Zip-14 can be neglected*: It has been shown that Zip-14 is a zinc influx transporter that mediates iron uptake along with zinc, and its expression is reciprocally and acutely modulated by cellular zinc status [53]. The culture medium used in these experiments (DMEM) does not contain zinc [54]. In addition, in FBS (used at 10% v/v) zinc concentration is negligible (0.1–1 *μ*M) compared to iron concentration (10–50 *μ*M) [55]. Hence, its activity can be neglected in these conditions.
6. *DMT1-mediated facilitated Fe*^2+^ *transport is uncoupled from H^+^*: DMT1’s mechanism is partially proton-coupled [26, 56]. *Fe*^2+^ uptake is considerably greater at pH 5-6 than at pH 7-8 [57]. Human DMT1 reaches its maximum activity at pH 6.75, which is equivalent to the pH of the BBM [58]. The H^+^ coupling feature increases DMT1’s affinity for *Fe*^2+^. However, at high extracellular pH, *Fe*^2+^ transport via DMT1 is not proton dependent [59]. To decouple iron transport from proton concentration, apical and basolateral media pH was set to 7 for all experiments. Caco-2 cells were cultured in a medium with sodium bicarbonate under a controlled CO_2_ atmosphere, which allowed keeping the pH practically constant despite the addition of ascorbic acid and perturbations associated to cell growth [55].

**Fig 2:**
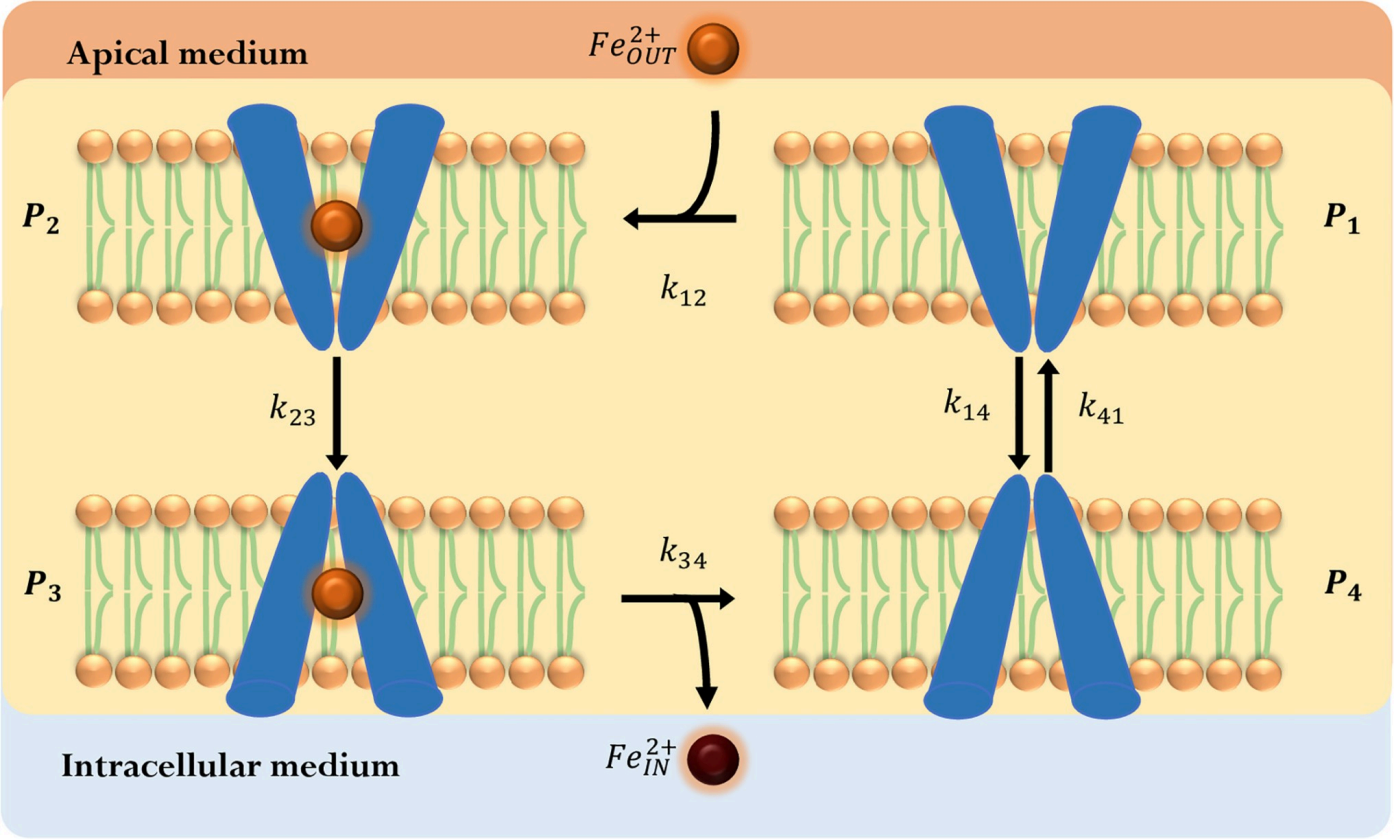
DMT1 phenomenological state-model. The model consists of a four state-cyclic representation of DMT1 kinetic mechanism. Empty DMT1 (*P*_1_) is oriented to the apical side where it can bind to *Fe*^2+^ (*P*_2_), then suffers a conformational change (*P*_3_), releases iron to the intracellular media (*P*_4_) and returns to its original state (*P*_1_). (orange circle): apical iron concentration ($Fe_{OUT}^{2+}$); (brown circle): iron concentration in the intracellular-basolateral space ($Fe_{IN}^{2+}$).

In this setting, DMT1 is the only relevant iron transport component that remains in the membrane and can interact with *Fe*^2+^. Therefore, we focus on a description of this transport system. To represent the main mechanisms of DMT1 that allow iron uptake, we propose a phenomenological model based on the mechanism presented in [27], as illustrated in Fig 2.

Iron binding (*P*_1_ → *P*_2_) happens in the high-pKa state of DMT1. After DMT1 binds to iron, a conformational change almost immediately occurs bringing the protein into an inward-open-like occluded state, where DMT1 flips over to a low-pKa conformation (*P*_2_ → *P*_3_). Iron is solvated and leaves DMT1’s binding site to become part of ferritin or cLIP (*P*_3_ → *P*_4_), and DMT1 recovers the high-affinity outward-open state (*P*_4_ → *P*_1_). In addition, iron concentration in the intracellular space near the apical membrane is too low to allow an interaction with DMT1 [60]. Based on this, only the transition *P*_1_ ↔ *P*_4_ is considered to be reversible.

Iron challenge induces the relocalization of DMT1 to intracellular domains into vesicles that allow the iron transport within the enterocyte. Thus, the number of DMT1 transporters in the apical membrane changes with time. Esparza *et al.* present evidence of the oscillatory behavior of DMT1 [61], quantifying the internalization of DMT1 from the apical membrane. While in the vesicle’s membrane, DMT1 can release iron from the vesicle to the cLIP [35, 36]. Although experimental information on the endocytosis process of the iron transporter is not abundant, to study its relevance, this process must be incorporated in the DMT1 state-model [25, 28, 32, 34].

In the following sections, two alternatives are presented to represent the oscillatory behavior of the DMT1 concentration over time. In these models, iron concentration inside the vesicles containing DMT1 is assumed to be constant and equal to the iron challenge concentration in the apical medium.

#### DMT1 binary switching-mechanism model

The repositioning mechanism of DMT1 allows regulating the intestinal iron absorption process. After iron feeding, iron transporters can be internalized from the apical membrane into vesicles within the intracellular compartment and then returned to the membrane.

**Fig 3:**
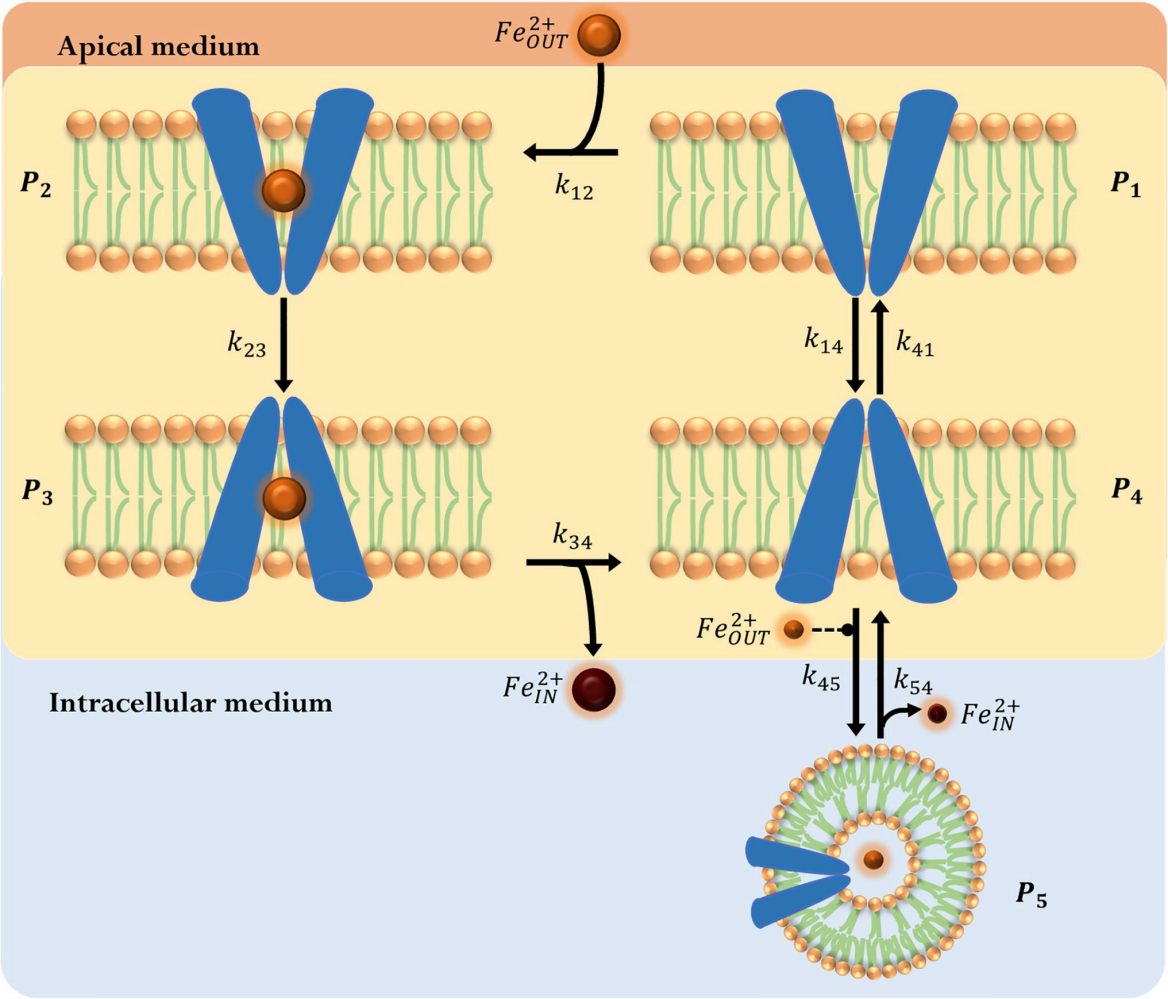
DMT1 binary switching-mechanism model. Considers DMT1’s endocyted state (*P*_5_). In this case, iron is released to the intracellular medium from the vesicle via DMT1. (orange circle): apical iron concentration ($Fe_{OUT}^{2+}$); (brown circle): iron concentration in the intracellular-basolateral space ($Fe_{IN}^{2+}$). Extracellular iron $Fe_{IN}^{2+}$ is considered to be a regulatory factor in endocytosis, represented as dashed modulator arrow (-- •).

Therefore, the decrease in the DMT1 concentration in the apical membrane due to its endocytosis can be considered a cyclic process. Although the internalization of DMT1 could occur for any state of DMT1 in Fig 2, the proposed model considers the internalization of *P*_4_ as representative of this process (Fig 3).

In the enterocyte, iron is transported by different mechanisms through vesicles containing DMT1. Under this assumption, the endocyted state of the transporter (*P*_5_) was added to the proposed phenomenological state-model.

A mathematical model comprising the differential equations derived from mass balances for all species in Fig 3, was obtained. This model considers all reactions in the mechanism to be elementary according to:

$$\begin{array}{ccc} \frac{dP_{1}}{dt} & = & -k_{12}P_{1}Fe_{OUT}^{2+}-k_{14}P_{1}+k_{41}P_{4}, \end{array}$$ (1)

$$\begin{array}{ccc} \frac{dP_{2}}{dt} & = & k_{12}P_{1}Fe_{OUT}^{2+}-k_{23}P_{2}, \end{array}$$ (2)

$$\begin{array}{ccc} \frac{dP_{3}}{dt} & = & k_{23}P_{2}-k_{34}P_{3}, \end{array}$$ (3)

$$\begin{array}{ccc} \frac{dP_{4}}{dt} & = & k_{34}P_{3}+k_{14}P_{1}-k_{41}P_{4}+k_{54}P_{5}-\left(k_{45}Fe_{OUT}^{2+}\right)P_{4}, \end{array}$$ (4)

$$\begin{array}{ccc} \frac{dP_{5}}{dt} & = & \left(k_{45}Fe_{OUT}^{2+}\right)P_{4}-k_{54}P_{5}, \end{array}$$ (5)

$$\begin{array}{ccc} \frac{dFe_{IN}^{2+}}{dt} & = & k_{34}DMT1_{E}P_{3}+\gamma k_{54}DMT1_{E}P_{5}, \end{array}$$ (6)

$$\begin{array}{ccc} \frac{dFe_{OUT}^{2+}}{dt} & = & -\frac{V_{cb}}{V_{a}}\cdot \frac{dFe_{IN}^{2+}}{dt}, \end{array}$$ (7)

In Eqs 1 to 7 the iron concentration in the intracellular-basolateral space and apical iron concentration are denoted by $Fe_{IN}^{2+}$ and $Fe_{OUT}^{2+}$, respectively. The amount of DMT1 in an average Caco-2 cell has not been reported, and it is assumed to be constant for the experimental time scale [32]. The proposed models do not consider the amount of DMT1 directly but an effective amount of transporter *DMT*1_*E*_ that is part of the set of model’s parameters, and corresponds to the ratio of the transporter concentration (DMT1) and the effectiveness of the model´s transport cycle Φ, $DMT1_{E}^{Model}=DMT1/\Phi_{Model}$. The factor Φ represents the effectiveness of iron transport and it is characteristic of the mathematical structure of each model.

*P*_*i*_ corresponds to the fraction of DMT1 in state *i* and *k*_*ij*_ the kinetic constant associated to the transition between states *i* and *j*. Eqs 1 to 3 are obtained directly by applying mass action law for all transitions involving these states; Eqs 4 to 6 have additional terms. Eq 7 is obtained by applying mass conservation. In Eqs 4 and 5 the term $\left(k_{45}Fe_{OUT}^{2+}\right)P_{4}$ accounts for the endocytosis of DMT1, which occurs only in the presence of an iron challenge. Eq 6 represents the iron uptake in the intracellular and basolateral space, as a result of direct transport by DMT1 in the *P*_3_ → *P*_4_ transition, and iron release from vesicles that are either fused with late endosomes or associated to PCBP2 [23]. Release from vesicles is represented by the term *γ k*_54_ *DMT1*_*E*_ *P*_5_, where *k*_54_ *DMT1*_*E*_ *P*_5_ is the maximum iron release rate associated to endocyted transporters, and *γ* is a volume correction factor that accounts for DMT1 containing vesicles that can transport iron to the intracellular media. This correction factor is estimated as the ratio between the volume of vesicles containing DMT1 that are capable of iron release *V*_*V*_ and the sum of the intracellular and basolateral volumes *V*_*cb*_. In enterocytes, the internal volume of vesicles totals 0.04 *μ*m^3^/*μ*m^2^ of the cellular surface area [62]. Considering this, the total area of enterocytes (*A*_*E*_, see Table 1) and assuming that 1-5% of vesicles are capable of iron release, the volume *V*_*V*_ is estimated to be 2.5 × 10^−10^ *μ*L [63–65].

**Table 1:** Cell culture characteristics.

Parameter	Description	Value	Unit
*h*_*c*_	Caco-2 height [66]	29.6	*μm*
*d*_*c*_	Caco-2 diameter [66]	6.2	*μm*
*R*_*V*_	Vesicles volume / cell surface area [62]	0.04	*μm*^3^/*μm*^2^
*V*_*a*_	Volume of apical medium [49]	200	*μ*L
*V*_*cb*_	Volume of cellular and basolateral medium [49]	1000	*μ*L
*V*_*m*_	Volume of the monolayer [49]	1.67	*μ*L
*V*_*V*_	Volume of vesicles containing DMT1	2.5 × 10^−10^*	*μ*L
*A*_*E*_	Enterocyte’s area	636.93**	*μm*^2^

*Calculated as *V*_*V*_ = *R*_*V*_⋅*A*_*E*_.

**Calculated assuming cylindrical shape, *A*_*E*_ = 2*π*(*d*_*c*_/2)*h*_*c*_ + 2*π*(*d*_*c*_/2)^2^.

To represent the oscillatory behavior of DMT1 in iron absorption, one of the simplest mathematical representations is to force the change of the rate constants of the *P*_4_ ↔ *P*_5_ reaction in time. To achieve this, the transporter’s endocytosis and its exocytosis processes are considered to occur in a mutually excluding manner, and to be dependent on DMT1’s amount on the apical membrane. It has been reported that in polarized iron starved Caco-2 cells, DMT1 is found primarily in the apical membrane, whereas in iron fed Caco-2 cells DMT1 undergoes endocytosis; in addition, the continuous presence of iron in the apical chamber allows internalization to continue until equilibrium is reached [25, 32]. After achieving equilibrium DMT1 returns to the apical membrane. Taking into account these facts, in the *Switch Model* we considered that DMT1’s endocytosis and its exocytosis processes can be modeled considering that these processes occur in a mutually excluding manner.

The endocytic mode is defined by setting *k*_45_ ≠ 0 and *k*_54_ = 0, and the exocytosis of DMT1 to the apical membrane of the enterocyte is defined by setting *k*_45_ = 0 and *k*_54_ ≠ 0. The model is initialized in the endocytic mode. Endocytosis occurs while the percentage of endocyted DMT1 is less than $\alpha_{DMT1}^{E}$. When this limit is exceeded, DMT1 exocytosis process is activated and remains active while the percentage of DMT1 in membrane is lower than $\alpha_{DMT1}^{M}$. When this limit is exceeded, the transporter endocytosis process is activated again.

#### DMT1 swinging-mechanism model

The oscillatory nature of biological processes has been observed and modeled in several biological systems. For instance, simple examples of cell cycle models can operate like autonomous oscillators [67], where (negative or positive) feedback loop of the components that interact in the biological system has the potential to generate self-sustained oscillations [68, 69]. In previous reports, it was confirmed that the positive feedback loop in oscillatory models increases the robustness of the oscillations in a greater number of conditions independently of the parameter values [70]. In addition, it has been shown that coupled positive and negative feedback loops are required in order to get oscillations [67]. The Belousov-Zhabotinsky reaction is the most thoroughly studied oscillating chemical reaction system [71]. Ball proposed a model to describe this reaction system, capable of exhibiting sustained oscillations over time, with a minimum number of components that interact through auto-catalytic reactions (governed by positive feedback) [72]. The series of competitive auto-catalytic reactions that define this system are: *A* + *B* → 2*A*, *B* + *C* → 2*B* and *C* + *A* → 2*C*, where the synthesis of *A* is autocatalyzed until the supply of *B* is depleted, *B* can only be produced if *C* is present, and *C* can only be produced if *A* is available, closing the auto-catalytic reactions circle. Initially, the following reaction is considered: *A* → *B*. This last reaction can be omitted if initially the species in the system are different from zero [73].

**Fig 4:**
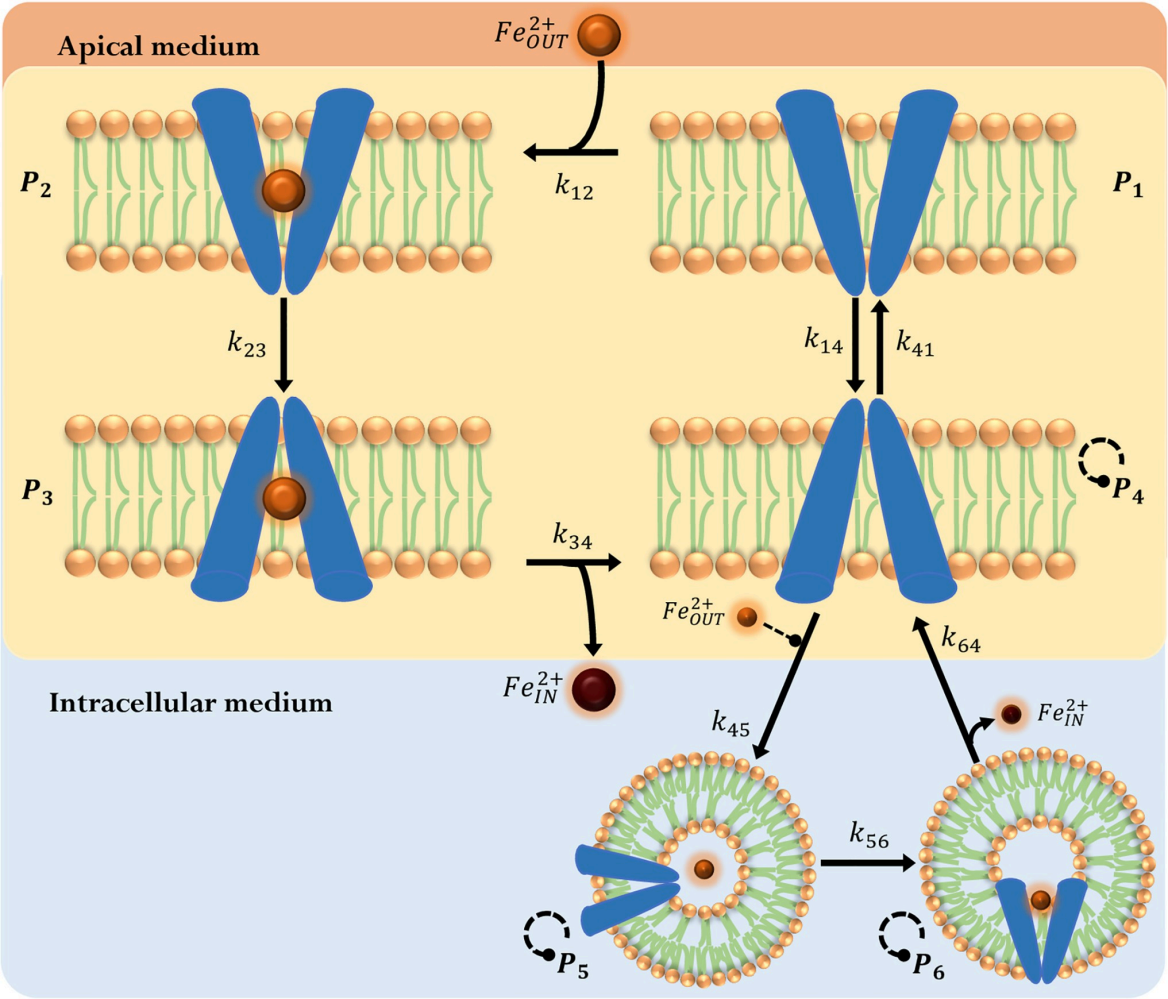
DMT1 swinging-mechanism model. DMT1 in the apical membrane unbound to iron and oriented to the intracellular side (*P*_4_) can be endocyted into iron containing vesicles (*P*_5_), where it can change to a state (*P*_6_) capable of iron release. (orange circle): apical iron concentration ($Fe_{OUT}^{2+}$); (brown circle): iron concentration in the intracellular-basolateral space ($Fe_{IN}^{2+}$). Extracellular iron $Fe_{IN}^{2+}$ is considered to be a regulatory factor in endocytosis, represented as dashed modulator arrow (-- •). The autocatalytic reactions of states *P*_4_, *P*_5_ and *P*_6_ is represented by a dashed circular modulator arrow.

When a model is coupled with an oscillator based on interlocked positive and negative feedback loops, the system can be capable of amplified oscillations sustained in time [73]. To capture the oscillatory nature of the iron uptake dynamics that has been observed experimentally [32], we propose a *DMT1 swinging-mechanism model*, where the DMT1 state-model presented above is coupled with the oscillator proposed by Ball. The main difference between this model and the *DMT1 binary switching-mechanism model* (*Switch Model*) is that the *DMT1 swinging-mechanism model* (*Swing Model*) allows for simultaneously occurring endocytosis and exocytosis mechanisms, representing the endocytosis process as the interplay of a set of auto-catalytic reactions. Both these models are capable of estimating $Fe_{IN}^{2+}$, $Fe_{OUT}^{2+}$ and the fraction of DMT1 located in the membrane. For simplicity, the interaction between these models is established as an interaction through the *P*_4_ state as shown in Fig 4. In this setting, components *A*, *C* and *B* of the Ball’s oscillator would correspond to states *P*_4_, *P*_5_ and *P*_6_ in the iron uptake model proposed. It is important to notice that in order for this oscillatory behavior to occur, initially, the fraction of DMT1 in species *P*_5_ and *P*_6_ must be different from zero.

A mathematical model comprising the differential equations derived from mass balances for all species in Fig 4, was obtained. This model considers all reactions in themechanism to be elementary according to:

$$\begin{array}{ccc} \frac{dP_{1}}{dt} & = & -k_{12}P_{1}Fe_{OUT}^{2+}-k_{14}P_{1}+k_{41}P_{4}, \end{array}$$ (8)

$$\begin{array}{ccc} \frac{dP_{2}}{dt} & = & k_{12}P_{1}Fe_{OUT}^{2+}-k_{23}P_{2}, \end{array}$$ (9)

$$\begin{array}{ccc} \frac{dP_{3}}{dt} & = & k_{23}P_{2}-k_{34}P_{3}, \end{array}$$ (10)

$$\begin{array}{ccc} \frac{dP_{4}}{dt} & = & k_{34}P_{3}+k_{14}P_{1}-k_{41}P_{4}+\rho k_{64}P_{6}P_{4}-\left(\rho k_{45}Fe_{OUT}^{2+}\right)P_{4}P_{5}, \end{array}$$ (11)

$$\begin{array}{ccc} \frac{dP_{5}}{dt} & = & \left(\rho k_{45}Fe_{OUT}^{2+}\right)P_{4}P_{5}-k_{56}P_{5}P_{6}, \end{array}$$ (12)

$$\begin{array}{ccc} \frac{dP_{6}}{dt} & = & k_{56}P_{5}P_{6}-\rho k_{64}P_{6}P_{4}, \end{array}$$ (13)

$$\begin{array}{ccc} \frac{dFe_{IN}^{2+}}{dt} & = & k_{34}DMT1_{E}P_{3}+\gamma k_{64}DMT1_{E}P_{6}, \end{array}$$ (14)

$$\begin{array}{ccc} \frac{dFe_{OUT}^{2+}}{dt} & = & -\frac{V_{cb}}{V_{a}}\cdot \frac{dFe_{IN}^{2+}}{dt}, \end{array}$$ (15)

Eqs 8 to 10 and 15 are the same equations used in the *Switch Model*. Eqs 11 to 14 have additional terms. The volume correction factor *γ* in Eq 14 is obtained as described above in the *Switch Model*. The term *ρ* in Eqs 11 to 13 is a kinetic correction factor applied to the reaction constants associated to the transporter’s endocytosis and exocytosis simultaneous processes. Without the term *ρ*, this model overestimates the rates of these processes after the second iron challenge leading to a higher apical iron uptake (over 20% of the experimental values, between minutes 6 and 9). On the other hand, when the kinetic correction factor is incorporated in the *Switch Model*, *ρ* increases the exocytocis rate in order to reach the conditions required for an endocytic switch thus decrease iron uptake. As a result, the addition of the term *ρ* to the *Switch Model* causes an overestimation in the prediction of $Fe_{IN}^{2+}$ between minutes 6 and 9 of the second iron challenge where exocytosis occurs.

It was reported that after an initial iron challenge, the uptake of a second iron dose decreases by approximately 33% compared to the first dose [32]. As a result, the total amount of iron absorbed by the cell at the end of a second dose would be 1.67 times the amount of iron absorbed in the first dose. We consider the kinetic correction factor *ρ* to be a function of time and the history of iron challenges to which the cells have been exposed, which allows damping the sustained oscillations of Ball’s model to represent the reduction in iron uptake observed as a result of mucosal block after a second iron dose. Biologically, this factor could be associated to regulation of transporters at a translational level, or to transporter degradation through the lysosomal degradation pathway [61]. Taking this into account we define *ρ* as follows:

$$\begin{array}{c} \rho \left(t,j\right)=1-2.8(\frac{Fe_{IN}^{2+}\left(t\right)-1.67Fe_{UP}^{2+}\left(j-1\right)}{Fe_{IN}^{2+}\left(t\right)}\cdot \frac{Fe_{CHG}^{2+}\left(j-1\right)}{Fe_{CHG}^{2+}\left(j\right)}), \end{array}$$ (16)

where *j* is the number of iron challenges to which the cells have been exposed, $Fe_{UP}^{2+}\left(j-1\right)$ is the amount of intracellular iron evaluated at time *t*_*j*_ just after the *jth* iron challenge is applied. $Fe_{CHG}^{2+}\left(j\right)$ is the iron concentration in the apical medium for the iron challenge *j*. $Fe_{UP}^{2+}$ and $Fe_{CHG}^{2+}$ are set to zero before the first challenge. The endocytosis of DMT1 is represented by the term $\left(\rho k_{45}Fe_{OUT}^{2+}\right)P_{4}P_{5}$ in Eqs 11 and 12 and, as in the previous model, it occurs only in the presence of an iron challenge when *P*_4_ and *P*_5_ states are non-zero. Finally, the term *ρk*_64_ *P*_6_ *P*_4_ in Eqs 11 and 13 accounts for the return of DMT1 to the apical membrane which, like in the case of Ball’s oscillator, can only occur if *P*_4_ and *P*_6_ states are non-zero.

#### Solution of the inverse problem

When experimental data cannot be easily obtained, the amount of data available to solve the inverse problem and fit the parameters of a model can be limited. In these cases, the possibility of overfitting is very clear as the number of experimental points is closer or exceeded by the number of parameters of the model [74]. Cross-validation is a technique for fitting model parameters, that involves repeated resampling of the full dataset until all data have been used in both training and testing, helping to avoid overfitting [75]. The leave-one-out cross-validation or Jackknife technique is the most commonly used resampling method to deal with limited data [74, 75]. The Jackknife parameter estimation technique is an iterative process, that is repeated as many times as the number of experimental observations available [76]. The parameters of the model are fitted to the experimental training dataset by minimizing the mean square error (*MSE*) between the element left out and the value predicted using the model, with the Nelder-Mead simplex algorithm [77]. At the end of the iterative process an estimate of the generalization error is obtained, given by the Jackknife *MSE* (*MSE*_*jk*_), which is defined as the sum of the differences between the experimental value removed in each iteration and the value predicted by the model. Both *Switch Model* and *Swing Model* parameters were obtained by minimizing *MSE* for the first iron challenge dataset. In addition for the *Swing Model* the estimation of the degree of mucosal block reported in the literature was considered [35]. Experimental data for the second challenge were used for phenomenologically validating the proposed model. We presented a detailed description of the application of the Jackknife method in [49]. It has been shown that the coefficient of determination *R*^2^ is an inadequate measure for the goodness of fit in non-linear models, since differences in model quality rarely affect its value more than in the third to fifth decimal place [78]. Akaike Information Criterion (AIC) is a measure widely accepted for determining the validity within a cohort of non-linear models and formulates the model selection problem as a search for the model with the lowest AIC value, simultaneously quantifying the precision and simplicity of the model [79, 80]. In this work, we assess the models using the bias-corrected Akaike Information Criterion (AICc) since we have a low number of experimental measurements. The model with lower AICc is selected as the most suitable model to characterize the experimental data.

#### Sensitivity analysis

Sensitivity Analysis (SA) methods allow studying how uncertainty in the output of a model can be associated to different sources of uncertainty in the model input factors [81]. This analysis can help verify and validate a model [82]. SA is a commonly used approach for identifying important parameters that exert great influence on a models’ behavior and quantitatively assess their effect [83].

The local approach to SA studies the impact that the variation of one parameter has on the model, while keeping the rest of parameters constant [84]. This deterministic approach consists in calculating the normalized local sensitivity coefficient *r*_*i*_, given by the first-order partial derivatives of outputs with respect to small changes in each parameter *θ*_*i*_, which allows a means of comparing sensitivities for input parameters that have different orders of magnitude [82, 84]. In the present study, $Fe_{IN}^{2+}$ is the model’s output and *r*_*i*_ is calculated as:

$$\begin{array}{c} r_{i}=\frac{\partial Fe_{IN}^{2+}/Fe_{IN}^{2+}}{\partial \theta_{i}/\theta_{i}}, \end{array}$$ (17)

For each model, one of its parameters was selected (*θ*_*i*_). Then, *r*_*i*_ was calculated to determine the variation of $Fe_{IN}^{2+}$ with respect to an increase ($r_{i}^{+}$) or decrease ($r_{i}^{-}$) of 10% in the nominal value of the chosen parameter *θ*_*i*_ and the largest value was selected ($r_{i}^{*}$). Based on this value, influence of each model’s parameter *θ*_*i*_ is classified as negligible for $|r_{i}^{*}|\leq 0.25$, low for $0.25<|r_{i}^{*}|\leq 0.5$; regular for $0.5<|r_{i}^{*}|\leq 1$; and high for $|r_{i}^{*}|>1$ [85].

#### Simulation details

Initial conditions for the *Switch Model* were set as $Fe_{IN}^{2+}\left(0\right)=0\mu M$, $Fe_{OUT}^{2+}\left(0\right)=20\mu M$, *P*_1_(0) = 1 and the rest of the fractions of DMT1 to be zero. For the *Swing Model* initial conditions were set as $Fe_{IN}^{2+}\left(0\right)=0\mu M$, $Fe_{OUT}^{2+}\left(0\right)=20\mu M$, *P*_1_(0) = 0.98, *P*_5_(0) = 0.01, *P*_6_(0) = 0.01 and the rest of the fractions of DMT1 to be zero. The initial guess for the optimization algorithm associated to the parameter fitting process was obtained from a preliminary study of the qualitative model behavior. All calculations and simulations were performed using MATLAB [86]. Both the training and test data sets are made available in S1 and S2 Tables in the Supplementary Information section. The source code is given in the S1 and S2 Appendices in the Supplementary Information section.

## Results and discussion

In the following sections, the performance of the proposed models is analyzed, comparing their predictive capabilities for the iron absorption process studied experimentally.

### Parameters estimation

The parameters of the proposed phenomenological models were fitted using the experimental data for iron uptake during the first challenge as a training set. The iron uptake data for the first 15 min after the second iron challenge was used as the validation set. In both models, the confidence intervals obtained for every parameter are narrow and they exhibit similar generalization errors, as shown in Tables 2 and 3.

**Table 2:** DMT1 binary switching-mechanism model parameters. Jackknife results for the first 15 minutes after iron exposure.

Parameter	Value	Unit	Confidence Intervals (*α* = 0.05)	*p-value*
*k*_12_	5.31 × 10^−6^	[*min*⋅*μM*]^−1^	±3.58 × 10^−7^	0.041
*k*_23_	0.9104	*min*^−1^	±0.0754	0.041
*k*_34_	2.2193	*min*^−1^	±0.2012	0.040
*k*_41_	0.3731	*min*^−1^	±0.0235	0.037
*k*_14_	11.2703	*min*^−1^	±0.6471	0.037
*k*_45_	0.0630	[*min*⋅*μM*]^−1^	±0.0027	0.043
*k*_54_	0.6492	*min*^−1^	±0.0421	0.039
*DMT*1_*E*_	566.10	*μ*M	±28.5262	0.043
$\alpha_{DMT1}^{E}$	0.9890	-	±0.0029	0.049
$\alpha_{DMT1}^{M}$	0.9780	-	±0.0278	0.046
*R*^2^ = 0.9273;*MSE*_*jk*_ = 1.0614;*AICc*_*Train*_ = 83.9573;*AICc*_*Test*_ = 84.0994				

**Table 3:** DMT1 swinging-mechanism model parameters. Jackknife results for the first 15 minutes after iron exposure.

Parameter	Value	Unit	Confidence Intervals (*α* = 0.05)	*p-value*
*k*_12_	1.68 × 10^−7^	[*min*⋅*μM*]^−1^	±5.08 × 10^−8^	0.0009
*k*_23_	1.6608	*min*^−1^	±0.1585	0.0008
*k*_34_	1.3221	*min*^−1^	±0.1284	0.0025
*k*_41_	0.7064	*min*^−1^	±0.0665	2.95 × 10^−9^
*k*_14_	30.7036	*min*^−1^	±0.3763	1.45 × 10^−8^
*k*_45_	0.2887	[*min*⋅*μM*]^−1^	±0.0018	1.06 × 10^−7^
*k*_56_	3.2082	*min*^−1^	±0.1273	7.41 × 10^−13^
*k*_64_	2.9639	*min*^−1^	±0.2923	2.15 × 10^−12^
*DMT*1_*E*_	46156.25	*μ*M	±615.9524	2.35 × 10^−8^
*R*^2^ = 0.9647;*MSE*_*jk*_ = 0.8256;*AICc*_*Train*_ = 61.6310;*AICc*_*Test*_ = 52.9433				

The model’s parameters are identified as significant at 95% confidence levels since all the *p-values* obtained are lower than 0.05 (t-test). The coefficient of determination calculated between each model and the experimental iron absorption data are shown in Tables 2 and 3. The high *R*^2^ values obtained in both cases indicate that the proposed models explain a high percentage of the experimental variance of the data considered.

Tables 2 and 3 show that $DMT1_{E}^{SwitchModel}>DMT1_{E}^{SwingModel}$. Considering the total mass of DMT1 as constant and equal in both models, then the effectiveness of the iron transport cycle for the *binary switching-mechanism model* (Φ_*Switch*_) is 80-fold larger than for the *swinging-mechanism model* (Φ_*Switch*_). The difference in the effectiveness factor between both models can be explained by the relative magnitudes of their respective parameters. For instance, *k*_12_ is ten times bigger in the *Switch Model*. Considering equal amounts of *P*_1_ and $Fe_{OUT}^{2+}$ in both models, this would translate in a larger iron entry to the transport cycle in the *Switch Model* compared to the *Swing Model*. In addition, the kinetic constant associated to the endocytosis process *k*_45_ is one order of magnitude smaller in the *Switch Model*. This favors the transport cycle for the *Switch Model* since a greater proportion of DMT1 would remain available for iron transport in the cycle compared to the amount endocyted. The parameter *k*_14_ related to the transporter conformational change process from *P*_1_ to *P*_4_ in the *Switch Model* is three times lower than in the *Swing Model*, indicating that at equal amounts of *P*_1_, a larger fraction of *P*_1_ would remain available for iron binding in the *Switch Model* than in the *Swing Model*.

### Sensitivity analysis

The sensitivity coefficient *r*_*i*_ was calculated to determine how an increase or decrease of each of the parameters affects the prediction of iron concentration in the intracellular-basolateral space for the two proposed models. This procedure was performed for all parameters in the *Switch Model* and *Swing Model*. Results of the sensitivity analysis for $Fe_{IN}^{2+}$ are shown in Tables 4 and 5.

**Table 4:** Parameters influence on $Fe_{IN}^{2+}$ for the *DMT1 binary switching-mechanism model*.

Parameter	Sensitivity coefficient $r_{i}^{+}$	Sensitivity coefficient $r_{i}^{-}$	Influence level
*k*_12_	0.9999	1.0001	High
*k*_23_	0.9997	0.9998	Regular
*k*_34_	0.9994	0.9995	Regular
*k*_41_	0.6534	0.6650	Regular
*k*_14_	-0.9008	-1.0992	High
*k*_45_	0.4852	0.4263	Low
*k*_54_	-0.4536	-0.4121	Low
*DMT*1_*E*_	1.0030	1.0000	High
$\alpha_{DMT1}^{E}$	-5.3340	-3.4025	High
$\alpha_{DMT1}^{M}$	3.7999	2.4144	High

**Table 5:** Parameters influence on $Fe_{IN}^{2+}$ for the *DMT1 swinging-mechanism model*.

Parameter	Sensitivity coefficient $r_{i}^{+}$	Sensitivity coefficient $r_{i}^{-}$	Influence level
*k*_12_	0.9999	1.0001	High
*k*_23_	0.9758	0.9789	Regular
*k*_34_	0.9792	0.9819	Regular
*k*_41_	0.6899	0.6905	Regular
*k*_14_	-0.9090	-1.1111	High
*k*_45_	-0.5734	-0.5747	Regular
*k*_56_	1.5265	1.5888	High
*k*_64_	0.4257	0.5340	Regular
*DMT*1_*E*_	1.0006	1.0007	High

The negative sensitivity coefficients shown in Tables 4 and 5 reflect that an increase in the parameter value is associated with a decrease in the model’s output ($Fe_{IN}^{2+}$) and vice versa [87]. Most parameters fall in the regular/high influence categories for both iron absorption models (Tables 4 and 5). This confirms that the mechanism stages considered in the proposed models are relevant for the description of the iron absorption process under study. The kinetic constants associated with the endocytosis of DMT1 (*k*_45_ in both models) and exocytosis of DMT1 to the apical membrane (*k*_54_ in Table 4 and *k*_64_ in Table 5), have the lowest influence on $Fe_{IN}^{2+}$. This indicates that small changes in these parameters have a reduced impact on the model’s output. Nevertheless, parameters $\alpha_{DMT1}^{E}$ and $\alpha_{DMT1}^{M}$ for the *Switch Model* and *k*_56_ for *Swing Model* were classified as highly influential on the value of $Fe_{IN}^{2+}$ predicted by the models. Both of these parameters are associated to the mathematical description of DMT1’s cycling process (endocytosis and exocytosis) in the models, indicating that DMT1’s localization is a crucial factor for the representation of intestinal iron absorption process in these models. The parameter *k*_12_ in both models has also a high influence on $Fe_{IN}^{2+}$ since it is associated with the binding of iron to empty DMT1 oriented to the apical side. The parameter *k*_14_ has a high influence in both models as it is associated to the only iron-independent entry to the endocytosis mechanism of the system, therefore allowing the modulation of the iron absorption process.

### Kinetics of mucosal block

**Fig 5:**
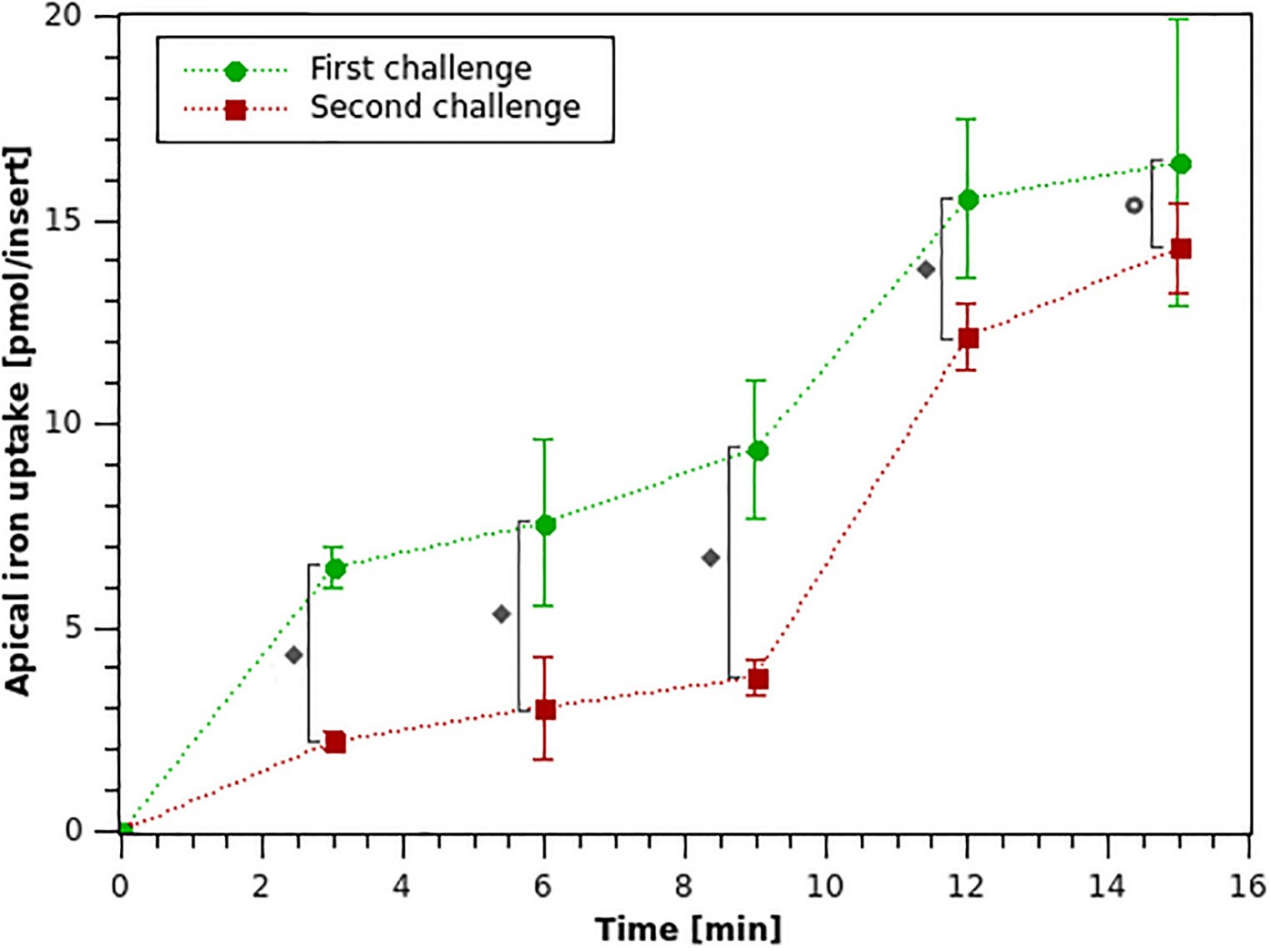
Apical iron uptake for Caco-2 cells after a first (0-15 min) and second (15-30 min) iron challenges with 20 *μ*M iron in the apical medium. (∘): the average value of the sample and error bars indicate its standard deviation. ♦ P < 0.05; ⊚ P < 0.25.

Results for iron uptake after the first and second iron challenges described in the methodology section, are shown in Fig 5.

It can be observed that the amount of apical iron absorbed is larger during the first iron exposure, which coincides with the mucosal block phenomenon that has been reported [32]. In fact, in the initial twelve minutes of the first challenge the amount of iron uptake is 16.4 pmol/insert, which is ∼1.4–fold the amount of iron absorbed in the second iron challenge. This difference is more noticeable in the first 9 minutes, where the amount of iron absorbed in the first challenge is ∼2.5–fold the amount of the second. Iron uptake was analyzed for both challenges from minutes 0 to 12 since a lower standard deviation was observed for both challenges in that time frame. A statistically significant difference between the iron uptake mean for the first and the second iron challenges was established using an independent-samples t-test to compare the means between the iron uptakes for each time point for both iron challenges, with a significance level of 5% for the first four time points (minutes 3-12), and 25% for the last time point (minute 15). The latter is due to the larger variability observed between experiments (see Fig 5), which is in agreement with what has been reported in literature for the variability of the Caco-2 experimental cell model [40]. The reduction in iron uptake between the first and second iron challenges ranges from 65.8% at minute 3 to 12.7% at minute 15. This is consistent with what has been reported in literature where a 33% reduction in iron uptake has been observed after an initial iron exposure in Caco-2 cells [35].

Considering that the lower variability for both challenges was obtained during the first three instants of time studied, iron uptake was analyzed for both challenges in that period. During the first challenge, in average only 9.4 pmol of iron were absorbed from the apical to the intracellular medium in the first nine minutes, which is equivalent to 0.47% of the initial iron dose (2000 pmol). Iron uptake is reduced in average during the second apical iron challenge where only 3.8 pmol were absorbed during the first nine minutes, which corresponds to 0.19% of the available iron in the apical membrane.

Changes in the observed iron absorption rate occur between the two iron challenges, suggesting the presence of non-linear components in the absorption process. For this reason, the iron absorption process cannot be effectively described using a Michaelis–Menten or Hill type expression [49]. The experimental patterns observed in the iron uptake rates over time could be attributed to the reduction in the amount of DMT1 present in the apical membrane after the iron exposure, as a result of the mucosal block suggested by Nuñez *et al.* [35].

### DMT1 binary switching-mechanism model

Cells were assumed to be in a cold induced constant metabolic state after the first challenge, as they were placed on ice and washed with cold PBS (see methodology); as a result, DMT1 states distribution would remain constant and all metabolic and transport processes would be halted. Therefore, the final state of the first challenge simulation is used as the initial condition for iron absorption after the second challenge.

**Fig 6:**
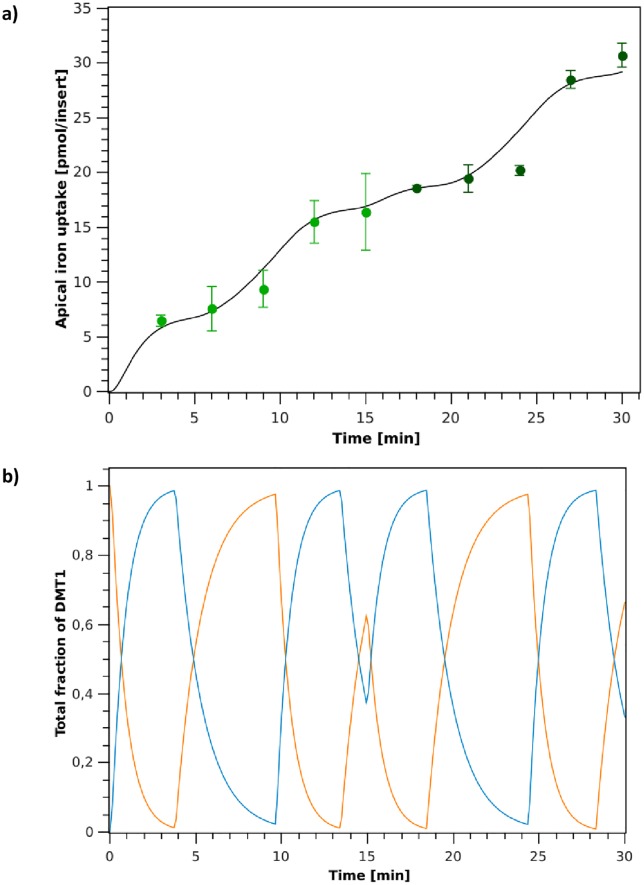
DMT1 binary switching-mechanism model simulation. **a**: Apical iron uptake after the first (0-15 min) and second (15-30 min) iron challenges. (∘): experimental data; (–): model simulation. **b**: Simulation of DMT1 endocytic cycling behavior during iron absorption for the first (0-15 min) and second (15-30 min) iron challenges. (orange line): Fraction of DMT1 in the apical membrane (*P*_1_ + *P*_2_ + *P*_3_ + *P*_4_); (blue line): Fraction of DMT1 in the endocyted state (*P*_5_).

*Switch Model* simulation of iron absorption fluxes and DMT1’s behavior over time, for the two iron challenges in the apical medium of the cells are shown in Fig 6. Simulations were performed using parameters shown in Table 2, which were determined for the experimental training set (first iron challenge data, 0-15 min). After the iron challenge, the model is initialized in the endocytic mode and validated with the experimental validation set (second iron challenge data, 15-30 min).

Simulation results in Fig 6a show the model’s capacity to represent the apical iron absorption fluxes observed experimentally for both, the training and validation datasets. The key feature of this proposed model is that it can capture the decrease in the rate of iron absorption when cells are exposed to a second iron challenge in the apical medium, effectively representing the mucosal block phenomenon. DMT1’s predicted distribution is shown in Fig 6b. Simulations capture the cycling behavior of DMT1 in the apical membrane (*P*_1_ + *P*_2_ + *P*_3_ + *P*_4_) and in its endocyted state (*P*_5_) due to internalization after iron exposure. For data points at 9 and 24 min the model overestimates iron uptake (see Fig 6a). This is due to the fact that, as shown in Fig 6b, at these time points most of transporters are located in the apical membrane. The characteristics of this *Switch Model* prevent DMT1’s distribution from reaching steady state, as the switch is activated when $\alpha_{DMT1}^{E}$ and $\alpha_{DMT1}^{M}$ are achieved (endocytic and exocytic switch, respectively). Fig 6b shows that the rate at which endocytosis of DMT1 occurs is greater than the rate of exocytosis of this transporter to the apical membrane, indicating that after iron exposure cells trigger DMT1’s internalization, and then return to the membrane through a slower process. This suggest that the model represents DMT1’s endocytic cycling process as a conservative control mechanism, where the control focus is placed on the return of transporters to the membrane.

The proposed *Switch Model* can predict the behavior of iron absorption fluxes obtained in our experiments. However, the model requires that DMT1’s endocytosis and its return to the membrane occurs in a mutually excluding manner, which may not be the best representation of these cellular processes. Therefore, a second phenomenological model is proposed to address this issue.

### DMT1 swinging-mechanism model

**Fig 7:**
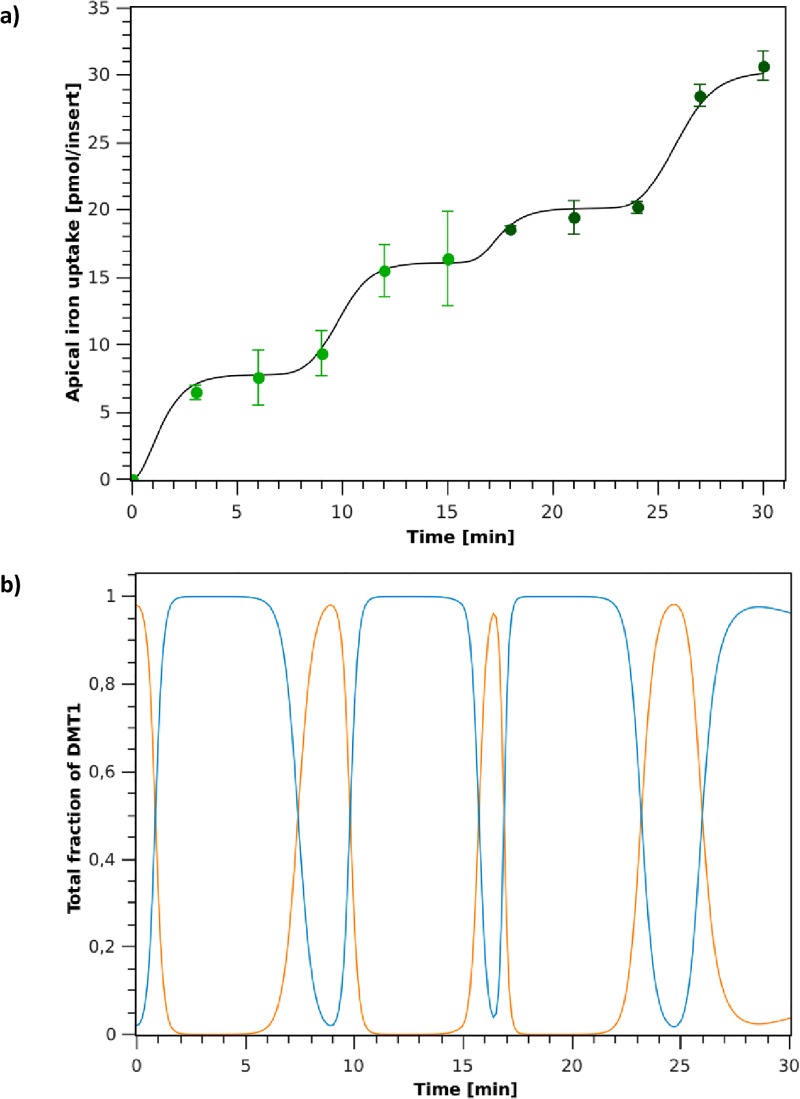
DMT1 swinging-mechanism model. **a**: Apical iron uptake after the first (0-15 min) and second (15-30 min) iron challenges. (∘): experimental data; (–): model simulation. **b**: Simulation of DMT1 endocytic cycling behavior during iron absorption for the first (0-15 min) and second (15-30 min) iron challenges. (orange line): Fraction of DMT1 in the apical membrane (*P*_1_ + *P*_2_ + *P*_3_ + *P*_4_); (blue line): Fraction of DMT1 in the endocyted state (*P*_5_ + *P*_6_).

*Swing Model* simulation of iron absorption fluxes and DMT1’s behavior over time, for the two iron challenges in the apical medium of the cells are shown in Fig 7. Simulations were performed using parameters shown in Table 3, which were determined for the experimental training set (first iron challenge data).

Simulation results show that the model is capable of representing the dynamics of the iron uptake in agreement with experimental data (Fig 7a). As in the previous case, the model captures the decrease in the iron absorption rate observed when cells are exposed to a second iron challenge in the apical medium, effectively representing the mucosal block phenomenon. However, unlike for the *Switch Model*, in this case the endocytosis and exocytosis processes can occur simultaneously. As a result, the proposed model captures iron uptake dynamics both qualitatively and quantitatively. As it can be observed in Fig 7b, this model maintains transporters in their endocyted state (*P*_5_ + *P*_6_) for longer periods of time. Once the transporter is endocyted, it remains in this state for approximately 3 minutes before returning to the apical membrane. On the other hand, as with *Switch Model*, the rate of return to the membrane of DMT1 is lower than its endocytosis rate. This strengthens the idea that iron uptake is a highly controlled processes.

The kinetic correction factor *ρ* is crucial for the model to effectively capture the reduction in iron absorption observed in the second challenge as it reduces the amount of DMT1 present in the apical membrane and the time interval that transporters remain in the membrane after iron feeding (see Fig 7b, min 15-17). This DMT1 relocalization is supported by reported experimental evidence, where it was observed that the transporter can remain endocitated for 40 minutes [25, 32]. At the end of the simulation (27-30 min) as shown in Fig 7b, the amplitude of the curve representing the endocyted state of the transporter (–) decreases with respect to the other cycles observed previously, indicating that a larger number of transporters remains in the apical membrane. This allows capturing the increase in the rate of iron absorption that occurs in this time interval.

### Final remarks

The phenomenological models generated, which take into account the main biological components of the system, allow capturing the complex iron uptake dynamics observed experimentally. Specifically, the models consider a description of DMT1 states and its endocytosis.

The internalization of DMT1 from the apical membrane as a result of iron feeding can substantially decrease intestinal iron absorption [25, 32]. The relatively fast kinetics of DMT1 relocalization could be a key element of the mechanism that regulates iron absorption in the short term, identified as a mucosal block, which would act as an earlier response than the transcriptional or translational regulations of DMT1 expression. Both *Switch Model* and *Swing Model* proposed in this study can represent DMT1’s endocytosis. But the second model allows DMT1 to remain in an endocyted state effectively blocking iron absorption after an iron challenge in the apical media. This second model would be in closer agreement with what has been reported for experimental observations by Nuñez *et al.* [35] where the reduction in iron uptake has been associated to decreased availability of DMT1 transporters in the membrane.

After an initial iron challenge, the amount of iron absorbed during the first 15 minutes is two orders of magnitude lower than the amount of iron present in the apical medium. It could be inferred then that extracellular iron remains practically constant and therefore, the difference in concentrations between the apical and intracellular media remains constant when media is replaced in the second challenge. This implies that, the reduction of the iron absorption rate observed for the second challenge is explained mainly by the oscillatory behavior of DMT1 concentration in the apical membrane.

Although the experiments were performed for short time intervals, mucosal block was observed, confirming that this fast regulatory mechanism modulates iron absorption in the short term. Our results also indicate that mucosal block acts in a time-scale of approximately 10 min after the second challenge, lower than what has been reported for this system [32]. This difference may be due to observation limitations in the experimental system.

Predictions obtained with the proposed models are in good agreement with the experimental data shown in Figs 6a and 7a, even with the validation data set which was not used for parameter estimation (15-30 min in Figs 6a and 7a). Hence, even though these may not describe the exact mechanism involved in the endocytosis process, which is not fully understood to date, they do capture the key characteristics of the biological phenomena observed experimentally in the apical iron absorption fluxes.

Although both models perform well in statistical terms, the *Swing Model* has a higher *R*^2^ and a lower *MSE*_*jk*_ and AICc (Table 3) than the *Switch Model* (Table 2), which is associated to its improved predictive capacity in the validation dataset. The main difference between these models is the incorporation of a Ball’s oscillator to represent the oscillatory behavior of DMT1s endocytosis. This mechanism would be in closer agreement to what occurs in the biological system than the *Switch Model* where endocytosis and exocytosis of DMT1 are considered to be mutually excluding, illustrating the fact that a better take on the mechanism involved in a biological process can significantly improve its mathematical representation.

Therefore, the *Swing Model* is the first phenomenological model reported to effectively represent the complexity of the iron absorption process, as it can predict the behavior of iron absorption fluxes after challenging cells with an initial dose of iron, and the reduction in iron uptake observed as a result of mucosal block after a second iron dose.

## Conclusion

Two phenomenological models based on a description of DMT1 states and its internalization were proposed to represent the iron uptake dynamics observed experimentally in Caco-2 cells: *binary switching-mechanism* and *swinging-mechanism DMT1 models*.

To describe the oscillatory behavior of the iron transporter, *Switch Model* considers that the DMT1’s endocytosis and return processes are mutually excluding. On the other hand, *Swing Model* couples DMT1 state-model with the Ball’s oscillator. Simulation results were compared with the experimental results, showing that both models were able to capture the oscillatory nature of the iron uptake dynamics determined experimentally, supporting the viability of the structure proposed.

Models simulations and experimental observations confirmed that iron uptake is a fast process and that the mucosal block is the fastest regulatory mechanism that acts to modulate this absorption process.

The *DMT1 swinging-mechanism model* is the first phenomenological model reported to effectively represent the complexity of the iron absorption process, as it can predict the behavior of iron absorption fluxes after challenging cells with an initial dose of iron, and the reduction in iron uptake observed as a result of mucosal block after a second iron dose.

## Supporting information

S1 Table Experimental training data of apical uptake over time for iron challenge concentration in the apical media of 20 *μ*M. (XLSX)

S2 Table Table Experimental test data of apical uptake over time for iron challenge concentration in the apical media of 20 *μ*M. (XLSX)

S1 Appendix Source code for *DMT1 binary switching-mechanism model*. a. Main program. b. Differential equations.
(TXT)

S2 Appendix Source code for *DMT1 swinging-mechanism model*. a. Main program. b. Differential equations.
(TXT)
